# A Novel Effector Gene *SCRE2* Contributes to Full Virulence of *Ustilaginoidea virens* to Rice

**DOI:** 10.3389/fmicb.2019.00845

**Published:** 2019-04-24

**Authors:** Anfei Fang, Han Gao, Nan Zhang, Xinhang Zheng, Shanshan Qiu, Yuejiao Li, Shuang Zhou, Fuhao Cui, Wenxian Sun

**Affiliations:** ^1^The Ministry of Agriculture Key Laboratory of Pest Monitoring and Green Management, and Joint Laboratory for International Cooperation in Crop Molecular Breeding, Ministry of Education, College of Plant Protection, China Agricultural University, Beijing, China; ^2^College of Plant Protection, Southwest University, Chongqing, China; ^3^College of Plant Protection, Jilin Agricultural University, Changchun, China

**Keywords:** effector, rice false smut, *Ustilaginoidea virens*, defense responses, fungal virulence

## Abstract

*Ustilaginoidea virens*, the causal agent of rice false smut (RFS), has become one of the most devastating rice pathogens worldwide. As a group of essential virulence factors, the effectors in the filamentous fungus might play central roles in the interaction between plants and pathogens. However, little is known about the roles of individual effectors in *U. virens* virulence. In this study, we identified and characterized a small secreted cysteine-rich effector, SCRE2, in *U. virens*. SCRE2 was first confirmed as an effector through yeast secretion, protein localization and translocation assays, as well as its expression pattern during *U. virens* infection. Transient expression of SCRE2 in *Nicotiana benthamiana* suppressed necrosis-like defense symptoms triggered by the mammalian BAX and oomycete elicitin INF1 proteins. The ability of SCRE2 to inhibit immunity-associated responses in *N. benthamiana*, including elicitor-triggered cell death and oxidative burst, is further defined to a small peptide region SCRE2^68-85^ through expressing a series of truncated proteins. Convincingly, ectopic expression of SCRE2 in the transgenic rice cells significantly inhibited pathogen-associated molecular pattern-triggered immunity including flg22- and chitin-induced defense gene expression and oxidative burst. Furthermore, the *scre2* knockout mutant generated by the CRISPR/Cas9 system greatly attenuated in *U. virens* virulence to rice. Collectively, this study indicates that the effector SCRE2 is able to inhibit plant immunity and is required for full virulence of *U. virens*.

## Introduction

The ascomycetous fungus *Ustilaginoidea virens* (Cooke) Takah (telemorph *Villosiclava virens*) is a biotrophic plant pathogen, which infects rice florets and thereby causes rice false smut (RFS) (Tanaka et al., 2008; Ashizawa et al., 2012; Tang et al., 2013; Hu et al., 2014; Zhang et al., 2014; Yong et al., 2018). With frequent outbreaks worldwide, RFS has recently become one of the most devastating rice diseases (Rush et al., 2000; Ladhalakshmi et al., 2012; Jecmen and Tebeest, 2015; Fan et al., 2016). *U. virens* colonizes rice florets and forms many false smut balls replacing filled grains on rice panicles, and thus causing a significant yield loss in this staple food crop. Besides, *U. virens* produces different types of mycotoxins, such as ustiloxins and ustilaginoidins in chlamydospore balls (Zhou et al., 2012). Seven ustiloxin and 26 ustilaginoidin derivatives have been identified and detected in the smut balls and/or in the mycelia so far (Zhou et al., 2012; Fu et al., 2017; Wang et al., 2017). These secondary metabolites, which are toxic to human and animals, reduce grain quality severely (Koyama et al., 1988; Luduena et al., 1994; Nakamura et al., 1994; Li et al., 1995; Shan et al., 2012; Wang et al., 2016; Fu et al., 2017). Recent studies illustrate infection processes of *U. virens*, including conidial germination on the surface of spikelet, mycelial growth, floral organ infection, and eventual false smut ball formation (Ashizawa et al., 2012; Tang et al., 2013; Hu et al., 2014; Fan et al., 2015; Yong et al., 2018). The infection life cycle of the pathogen includes both sexual (ascospores) and asexual (chlamydospores) stages (Zhang et al., 2014).

With the help of recently-released *U. virens* genome (Zhang et al., 2014), several pathogenicity factors have recently been identified through screening of a T-DNA insertion mutant library in *U. virens. UvSUN2* encoding a SUN family protein was found to be probably required for fungal growth, cell wall construction, stress response and virulence in *U. virens* (Yu et al., 2015). *UvPRO1* was also identified to regulate conidiation, stress response, and virulence of *U. virens* (Lv et al., 2016). In contrast, the knockout of *Uvt3277*, encoding a low-affinity iron transporter protein, caused increased virulence in *U. virens* (Zheng et al., 2017).

As a group of important virulence factors, pathogen effectors play central roles in the host-pathogen interactions (Dou and Zhou, 2012). Adapted pathogens secrete a large array of effectors to inhibit pathogen-associated molecular pattern (PAMP)-triggered immunity (PTI), which is initiated after the perception of PAMPs by pattern recognition receptors (PRRs) in plants (Macho and Zipfel, 2014). Evolutionarily, another type of immune responses, effector-triggered immunity (ETI), usually accompanied by the hypersensitive response (HR), is activated in plant cells by resistance (R) proteins specifically recognizing certain pathogen effectors or their actions (Jones and Dangl, 2006; Stergiopoulos and de Wit, 2009; Chae et al., 2016; Peng et al., 2018).

Ever-increasing numbers of effectors in phytopathogenic fungi, oomycetes, bacteria and nematodes were found to manipulate plant innate immunity through different molecular strategies (Lo Presti et al., 2015; Chae et al., 2016). For example, Slp1 from *Magnaporthe oryzae* and Ecp6 from *Cladosporium fulvum* can perturb host chitin-triggered immunity by sequestering chitin oligosaccharides through LysM domains to block chitin binding to its receptors (de Jonge et al., 2010; Mentlak et al., 2012; Sanchez-Vallet et al., 2013). The *Ustilago maydis* effector Pit2 serves as an inhibitor of a set of apoplastic maize cysteine proteases and is essential for fungal virulence (Mueller et al., 2013). Another secreted effector Pep1 (Protein essential during penetration-1) also plays an essential role in *U. maydis* virulence and inhibits oxidative burst driven by the secreted maize peroxidase POX12 (Hemetsberger et al., 2012, 2015). A lipase effector FGL1 in *Fusarium graminearum* releases free fatty acids to prevent immunity-related callose formation during wheat head infection, and therefore, the Δ*fgl1* mutant has a limited ability to infect wheat spikelets (Blumke et al., 2014).

On the other hand, many effectors or their actions are perceived by R proteins and thus induce plant resistance (Selin et al., 2016). For example, AvrLm1 to AvrLm9 from *Leptosphaeria maculans* are recognized by Rlm1 to Rlm9, respectively, causing incompatible interaction between the pathogen and canola plants (Balesdent et al., 2002). Interestingly, the apoplastic effector XEG1 in *Phytophthora sojae*, a member of glycoside hydrolase family 12, not only contributes to pathogen virulence, but is also recognized as a PAMP by the receptor RXEG1 to induce cell death in dicot plants (Ma et al., 2015; Wang et al., 2018). PsXLP1, a PsXEG1 paralog that loses the enzyme activity, binds to GmGIP1 more tightly than PsXEG1, and thus releasing PsXEG1 to promote virulence (Ma et al., 2017). In addition, multiple effectors have been identified to induce plant immunity in non-host plants (Chen et al., 2013; Fang et al., 2016).

A total of 193 small cysteine-rich secreted proteins have been considered as putative effectors in *U. virens*. The HR inhibitory assays in *N. benthamiana* leaves, together with transcriptome analyses during different stages of *U. virens* infection, suggest that the majority of putative effectors function to manipulate plant immune responses for successful colonization in *U. virens* (Zhang et al., 2014). Meanwhile, several putative effectors in *U. virens* have been also identified to induce cell death or defense responses in the non-host *N. benthamiana* and host rice. Similar to cell death-inducing *M. oryzae* effectors (Chen et al., 2013), signal peptides (SPs) of these *U. virens* effectors are essential for their abilities to induce cell death (Fang et al., 2016). Collectively, many putative effectors in *U. virens* most likely have the ability to suppress or activate plant defenses. However, few effectors have been confirmed and identified in *U. virens* and the functions of individual effectors are largely unknown.

It has been previously demonstrated that UV_1261, small Secreted Cysteine-Rich Effector candidate 2 (named hereafter as SCRE2) with 130 amino-acid residues, can suppress non-host cell death triggered by *Burkholderia glumae* in *N. benthamiana* (Zhang et al., 2014). In this study, SCRE2 is determined to be an effector through yeast secretion and cell translocation assays. We further demonstrated that SCRE2 suppresses different types of immune responses in non-host *N. benthamiana* and in host rice. Interestingly, a small peptide region SCRE2^68-85^ retains the ability to inhibit cell death and oxidative burst in *N. benthamiana*. Importantly, the *scre2* mutant is attenuated in virulence to rice. Collectively, we showed that SCRE2 is an important effector that is required for full virulence of *U. virens*.

## Results

### SCRE2 Is an Effector in *U. virens*

**FIGURE 1:**
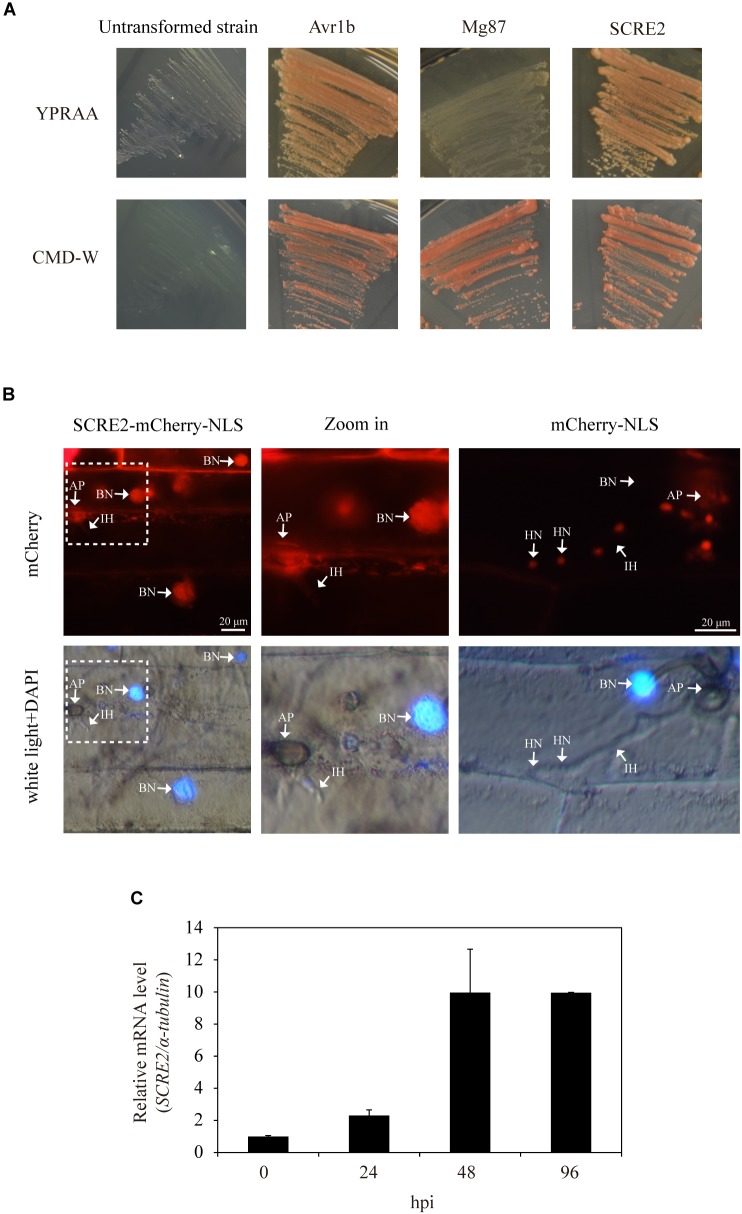
SCRE2 is an effector in *U. virens* revealed by yeast secretion and cell translocation assays. **(A)** Functional validation of the signal peptide of SCRE2 by yeast secretion assay. The *SCRE2^SP^-SUC2*-transformed YTK12 yeast strain was able to grow on YPRAA medium plates with raffinose as sole carbon source. The signal peptide of Avr1b in *P. sojae* and the N-terminal sequences of Mg87 in *M. oryzae* were used as positive and negative controls, respectively. The un-transformed YTK12 did not grow on either CMD-W or YPRAA media. Lower panel, yeast growth on CMD-W media showed an equal viability of different transformed strains. **(B)** The cell translocation assay of SCRE2. The expression of SCRE2-mCherry-NLS in conidia of the transformed *M. oryzae* P131 strain was confirmed by red fluorescence under fluorescence microscopy (in Supplementary Figure S2). Barley leaves were inoculated with the transformed strains and were then incubated in a moist, dark chamber at 28°C. *M. oryzae*-infected barley lower epidermal cells were observed at 30 h post-inoculation (hpi) via fluorescence microscopy. DAPI staining showed cell nuclei. NLS, nuclear localization signal; IH, infectious hyphae; AP, appressoria; BN, barley nuclei; HN, hyphal nuclei. **(C)** Expression pattern of *SCRE2* during *U. virens* infection of the susceptible rice cultivar LYP9. The *U. virens*-inoculated panicles of the cultivar LYP9 were collected at 0, 24, 48 and 96 h post-infiltration (hpi) for gene expression analyses using quantitative real time RT-PCR assays. Data are means ± standard errors (SE). The α*-tubulin* gene was used as an internal reference.

*SCRE2* encodes a putative secreted protein with 130 amino acid residues (Supplementary Figure S1). A yeast secretion system has been developed to validate the functionality of the predicted SP (Jacobs et al., 1997; Fang et al., 2016). The predicted SP-encoding sequence of *SCRE2* was fused in frame with the truncated *SUC2* gene encoding an invertase without its own SP. The fusion construct was transformed into the invertase secretion-deficient yeast strain YTK12 that is unable to utilize raffinose as carbon source. We observed that the *SP^SCRE2^-SUC2*-transformed YTK12 strain grew on YPRAA medium with raffinose as sole carbon source (Figure 1A), indicating that the fusion protein SP^SCRE2^-SUC2 is successfully secreted into the medium and degrades raffinose into simple sugars to support YTK12 growth. SP^Avr1b^ and the N-terminal peptide of Mg87 were also expressed as a fusion with the truncated SUC2 in YTK12 as positive and negative controls, respectively (Figure 1A). The results indicate that the predicted SP of SCRE2 is functional to direct the protein to the secretory pathway.

Subsequently, living cell imaging was performed to investigate whether SCRE2 is secreted and translocated into plant cells during infection (Park et al., 2012). The mCherry-labeled SCRE2 carrying a nuclear localization signal (NLS) from simian virus large T-antigen was ectopically expressed under the control of the RP27 promoter in the *Magnaporthe oryzae* isolate P131 (Peng and Shishiyama, 1988). The expression of SCRE2 tagged by red fluorescent protein mCherry was detectable in the conidia of the engineered *M. oryzae* strain (Supplementary Figure S2). When the strain was inoculated onto detached barley leaves, the majority of *M. oryzae*-infected epidermal cells exhibited red fluorescence in the nuclei indicated by DAPI staining at 30 h after inoculation (Figure 1B). As a negative control, red fluorescence was observed in the nuclei of infected hyphae, but not in barley cell nuclei when barley leaves were inoculated with the transformed *M. oryzae* strain expressing mCherry-NLS (Figure 1B). Together, these data indicate that SCRE2 is secreted and translocated inside plant cells during pathogen infection.

### *SCRE2* Is Transcriptionally Up-Regulated During *U. virens* Infection

In order to reveal the expression pattern of *SCRE2* during *U. virens* infection, the highly virulent isolate P1 was artificially inoculated into young panicles of the rice cultivar LYP9, which is highly susceptible to P1 (Han et al., 2015). Quantitative real time RT-PCR showed that *SCRE2* expression was up-regulated gradually during early stages of infection (Figure 1C). The expression level was increased by ∼2–10-fold at different infection stages. The results indicate that SCRE2 may play a role in the interaction of rice and *U. virens*.

### *SCRE2* Is Highly Conserved in *U. virens* Isolates

Positive selection pressure from host resistance genes has a marked impact on the evolution of effector genes in filamentous plant pathogens (Raffaele and Kamoun, 2012). Therefore, fungal effectors generally share a relatively low amino acid sequence similarity among closely-related fungal species. Consistently, we found that the putative effectors in *U. virens* and *Metarhizium anisopliae* are highly diverse despite their close evolutionary relationship (Zhang et al., 2014). Through Pfam and BLAST searches, neither homolog nor similar functional motif/domain of SCRE2 was found in closely-related fungal species. To investigate sequence conservation of SCRE2 in *U. virens*, *SCRE2* was PCR-amplified from 29 Chinese isolates collected from different regions, including Guangdong, Jiangsu, Yunnan, Shandong, Hunan, Anhui, Liaoning and Henan Provinces and was then subject to sequencing. Multiple sequence alignment analysis showed that SCRE2 protein sequences were all identical in the 29 isolates and four genome-sequenced strains from China, Japan, India, and United States (Kumagai et al., 2016; Devanna et al., 2018), suggesting that SCRE2 is highly conserved in *U. virens* (Supplementary Figure S3 and Supplementary Table S1).

### SCRE2 Is Localized in Periplasmic Spaces and Perhaps in Multiple Subcellular Compartments

**FIGURE 2:**
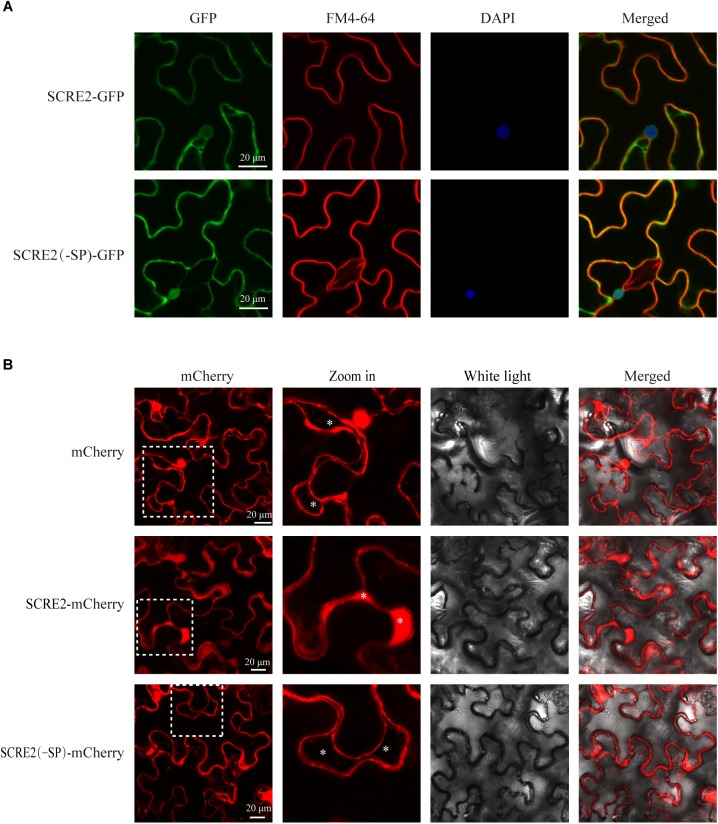
SCRE2 is localized in multiple subcellular compartments and periplasmic spaces in plants. **(A)** SCRE2-GFP was localized to the nuclei, cytoplasm, and plasma membrane of *N. benthamiana* cells. Green fluorescence was observed in the *N. benthamiana* cells transiently expressing SCRE2-GFP and SCRE2(-SP)-GFP via laser scanning confocal microscopy. GFP panels: green fluorescence; FM4-64 panels: red fluorescence after staining with the membrane-binding lipophilic dye FM4-64; DAPI panels: blue fluorescence in the nuclei after staining with DAPI; merged panels: overlay images of the three fluorescence signals. The yellow color in overlay images indicates overlap of green and red fluorescence, while the cyan color in overlay images indicates overlap of green and blue fluorescence. **(B)** SCRE2-mCherry was localized in the periplasmic spaces of *N. benthamiana* cells after plasmolysis. Red fluorescence was observed in the plasmolyzed *N. benthamiana* cells transiently expressing mCherry, SCRE2-mCherry, and SCRE2(-SP)-mCherry via laser scanning confocal microscopy. SCRE2(-SP): SCRE2 without the signal peptide; mCherry panels: red fluorescence; Zoom in panels: enlarged images from broken square areas in mCherry panels. Astericks indicate the periplasmic spaces after *N. benthamiana* cells were plasmolyzed. Scale bar, 20 μM.

In order to determine subcellular localization of SCRE2, the protein was expressed in fusion with GFP and mCherry driven by the 35S promoter in *N. benthamiana* leaves via *Agrobacterium*-mediated transient expression. Green fluorescence was observed in multiple subcellular compartments of the infiltrated *N. benthamiana* cells (Figure 2A). The cells were also stained with the membrane-binding amphiphilic dye FM4-64 and the DNA stain 4′, 6-diamidino-2-phenylindole (DAPI). Red fluorescence from FM4-64 was partially overlapped with green fluorescence, indicating that SCRE2 is not only localized to the membrane, but also in the cytoplasm. Meanwhile, DAPI staining further showed that SCRE2-GFP is present in the nuclei of *N. benthamiana* cells (Figure 2A). We also demonstrated that the recombinant SCRE2-GFP protein without the SP had a similar localization pattern after being transiently expressed in the leaves of *N. benthamiana*. Transient expression of SCRE2-GFP and SCRE2(-SP)-GFP in rice protoplasts exhibited a similar subcellular localization in the nuclei and cytoplasm, and on the membrane as well. Co-expression of mCherry-NLS confirmed a nuclear localization of SCRE2-GFP and SCRE2(-SP)-GFP in rice protoplasts (Supplementary Figure S4A). Western blot analyses showed that SCRE2-GFP and SCRE2(-SP)-GFP were stably expressed in *N. benthamiana* cells and in rice protoplasts (Supplementary Figures S4B,C). However, precise localization of SCRE2 needs to be further elucidated since free GFP was also localized to cell nuclei (Supplementary Figure S4A). Furthermore, SCRE2-mCherry and SCRE2(-SP)-mCherry were expressed in *N. benthamiana* cells that were subsequently subject to plasmolysis. SCRE2-mCherry was clearly observed in periplasmic spaces, while SCRE2(-SP)-mCherry and mCherry were not present in periplasmic spaces (Figure 2B). Expression of mCherry-labeled proteins was also verified via immunoblotting analysis (Supplementary Figure S4D). The observation indicates that SCRE2 is secreted by plant cells. Therefore, SCRE2 is localized in periplasmic spaces and perhaps in multiple subcellular compartments of plant cells.

### SCRE2 Suppresses BAX- and INF1-Induced Cell Death in *N. benthamiana*

**FIGURE 3:**
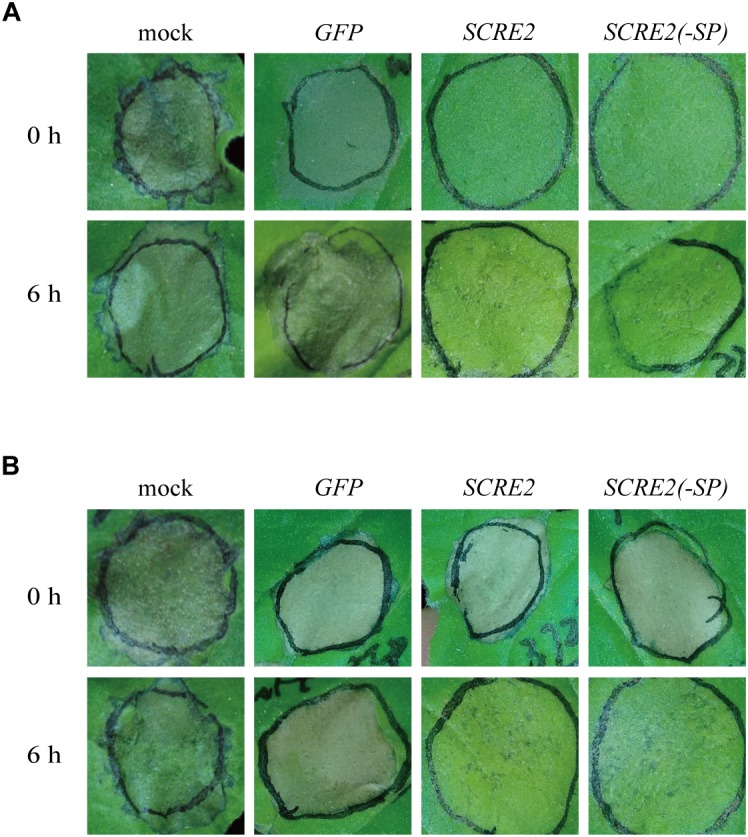
SCRE2 suppresses BAX- and INF1-triggered cell death in *N. benthamiana.* Expression of BAX **(A)** and INF1 **(B)** in *N. benthamiana* leaves alone triggered necrosis-like immunity symptoms (mock). Transiently expressed SCRE2 and SCRE2(-SP) suppressed cell death triggered by BAX **(A)** and INF1 **(B)** in *N. benthamiana* leaves. The *A. tumefaciens* strain carrying the *SCRE2* gene construct was coinfiltrated with *A. tumefaciens* cells carrying *BAX* **(A)** and *INF1* **(B)** gene constructs either simultaneously (upper panels) or 6 h prior to infiltration (lower panels) into *N. benthamiana* leaves. *SCRE2(-SP)*: *SCRE2* without the signal peptide. Cell-death symptoms on the infiltrated leaves were photographed at 3 days after the final infiltration. *A. tumefacien* strains carrying *GFP* or *SCRE2(-SP)* were infiltrated into *N. benthamiana* leaves using the same procedure. *SCRE2(-SP)*: the *SCRE2* gene lacking DNA sequences encoding the signal peptide.

Cell death symptoms triggered by the pro-apoptotic mouse protein BAX and the *Phytophthora infestans* elicitin INF1 in plants physiologically resemble those associated with the immunity-related hypersensitive response (Lacomme and Santa, 1999; Wang et al., 2011; Figure 3). Therefore, the ability to inhibit BAX- and INF1-triggered cell death in *N. benthamiana* has proved to be a valuable judgment for the immunosuppressive ability of pathogen effectors (Dou et al., 2008a; Chen et al., 2018). Here, *Agrobacterium* strains carrying either *SCRE2* or *BAX* gene construct were mixed and co-infiltrated into *N. benthamiana* leaves. Alternatively, SCRE2 was expressed in *N. benthamiana* at 6 h prior to infiltration of the *Agrobacterium* strain carrying *BAX* gene construct. It was demonstrated that transiently expressed SCRE2 suppressed BAX-triggered cell death in both situations (Figure 3A). Similarly, we also determined the ability of SCRE2 to suppress necrosis-like immunity symptoms in *N. benthamiana* triggered by INF1. Interestingly, SCRE2 suppressed INF1-triggered cell death when it was expressed at 6 h before INF1 expression, while simultaneous expression of SCRE2 did not inhibit INF1-triggered cell death (Figure 3B). As a negative control, GFP expression did not suppress BAX- and INF1-triggered necrosis-like symptoms in *N. benthamiana* (Figure 3). Together with the previous finding that SCRE2 inhibits *B. glumae* induced non-host cell death (Zhang et al., 2014), these data indicate that SCRE2 suppresses different types of immunity-associated responses in *N. benthamiana*.

### Functional Domain Analysis of SCRE2

**FIGURE 4:**
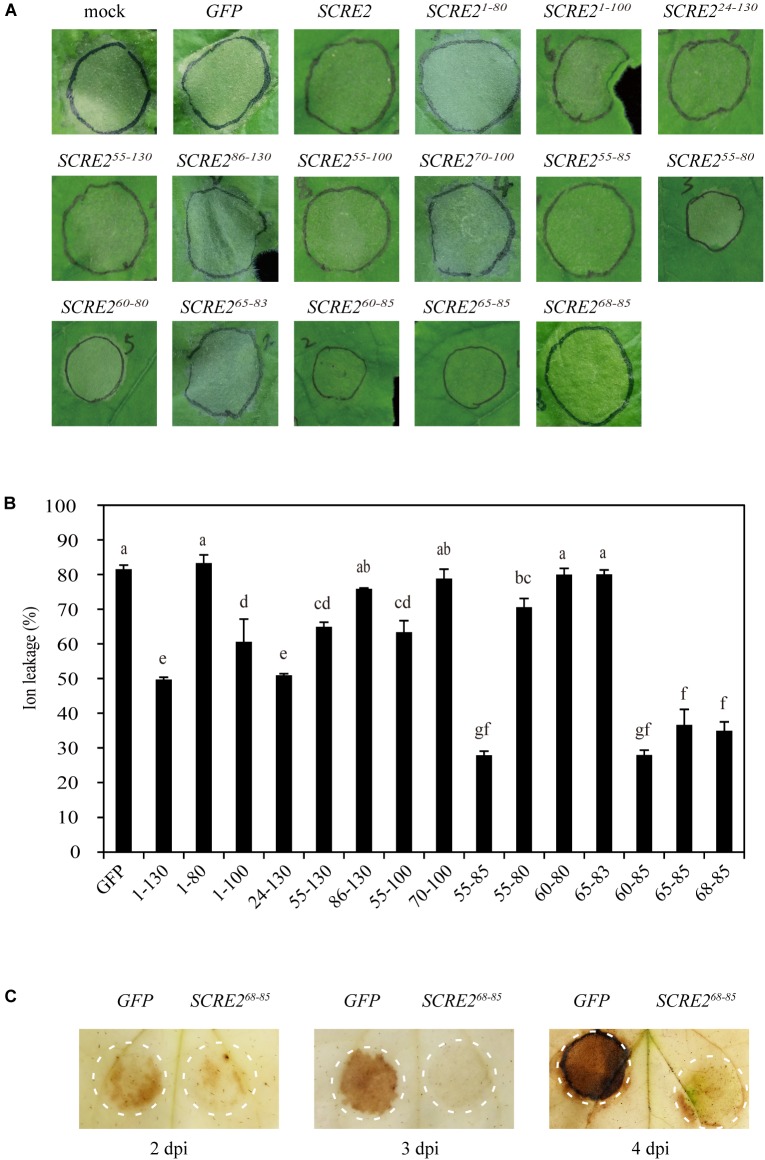
SCRE2^68-85^ is defined as a small peptide region to retain the immunosuppression ability in *N. benthamiana*. **(A)** cell-death symptoms on the infiltrated leaves at 4 days after co-infiltration with *Agrobacterium* strains carrying the *BAX* gene and different *SCRE2* constructs expressing SCRE2, SCRE2^1-80^, SCRE2^1-100^, SCRE2^24-130^, SCRE2^55-130^, SCRE2^86-130^, SCRE2^55-100^, SCRE2^70-100^, SCRE2^55-85^, SCRE2^55-80^, SCRE2^60-80^, SCRE2^65-83^, SCRE2^60-85^, SCRE2^65-85^, and SCRE2^68-85^, respectively. **(B)** The severity of cell death was quantified by ion leakage in the infiltrated *N. benthamiana* leaves. Ion leakage from leaf discs co-infiltrated with *Agrobacterium* strains carrying the *BAX* gene and different truncated *SCRE2* constructs was measured at 4 days post-infiltration (dpi). Co-expression of green fluorescent protein (GFP) was used as a negative control. Data are means ± standard error (SE) from three independent experiments. Different letters (a–g) indicate significant difference in ion leakage from leaf discs co-expressing *BAX* and different truncated *SCRE2* proteins (*P* < 0.05). **(C)** BAX-triggered reactive oxygen species (ROS) accumulation in the infiltrated *N. benthamiana* leaves was largely attenuated by co-expression with SCRE2^68-85^ as compared with GFP co-expression. The *Agrobacterium* strain carrying *GFP* or *SCRE2^68-85^* construct was coinfiltrated into *N. benthamiana* leaves with *Agrobacterial* cells carrying the *BAX* gene construct, respectively. 3, 3′-diaminobenzidine (DAB) staining was performed at 2, 3, and 4 dpi. Circles represent the infiltrated areas in the leaves.

To determine whether small peptide regions in SCRE2 are functional to inhibit BAX-induced cell death, multiple truncated proteins of SCRE2, such as the N-terminal SCRE2^1-80^ and SCRE2^1-100^, the C-terminal SCRE2^24-130^, SCRE2^55-130^ and SCRE2^86-130^, and the middle regions, SCRE2^55-100^, SCRE2^70-100^ and SCRE2^55-85^ were first co-expressed with BAX in *N. benthamiana* (Figure 4). HR inhibitory assays showed that the first 80 amino-acid regions (SCRE2^1-80^), the C-terminal SCRE2^86-130^ and the middle peptide region SCRE2^70-100^ completely lost the ability to inhibit BAX-triggered cell death. In contrast, other tested peptides retained the ability to suppress BAX-triggered cell death. In particular, the small peptide region SCRE2^55-85^ had an even stronger ability to suppress cell death (Figure 4A). To delineate the smaller region with immunosuppressive ability in SCRE2, more truncated constructs expressing SCRE2^55-80^, SCRE2^60-80^, SCRE2^65-83^, SCRE2^60-85^, SCRE2^65-85^, and SCRE2^68-85^ were generated. Transient expression of 18-amino-acid small peptide SCRE2^68-85^ was still able to inhibit cell death (Figure 4A). Ion leakage is generally accompanied with cell death and is often used to quantify the degree of cell necrosis (Mittler et al., 1999). We showed that the observed cell-death symptoms were highly correlated with the percentage of electrolyte leakage in *N. benthamiana* leaves (Figure 4A,B). Consistently, ion leakage from *N. benthamiana* leaves co-expressing BAX and small truncated peptides SCRE2^55-85^, SCRE2^60-85^, SCRE2^65-85^, and SCRE2^68-85^ was significantly lower than that from SCRE2-expressing leaves, indicating that these peptides have an even stronger ability to suppress BAX-induced cell death than the full-length protein. As a negative control, *N. benthamiana* leaves co-expressing BAX and GFP exhibited the highest level of ion leakage (Figure 4B). Immunoblotting analyses showed that some larger truncated proteins were well expressed, while small peptides (∼3 kDa) were not detected probably due to technical reasons (Supplementary Figure S5). To investigate how SCRE2 suppresses cell-death symptoms, we detected BAX-triggered reactive oxygen species (ROS) generation in *N. benthamiana* leaves via DAB staining. The results showed that SCRE2^68-85^ expression strongly suppressed ROS generation triggered by BAX compared with GFP at 2, 3, and 4 dpi (Figure 4C). Together, the results suggest that SCRE2^68-85^ defines a relatively small peptide region that retains the ability to suppress immunity-associated responses.

### Ectopic Expression of SCRE2 in Transgenic Rice Cells Suppresses Rice Immunity

**FIGURE 5:**
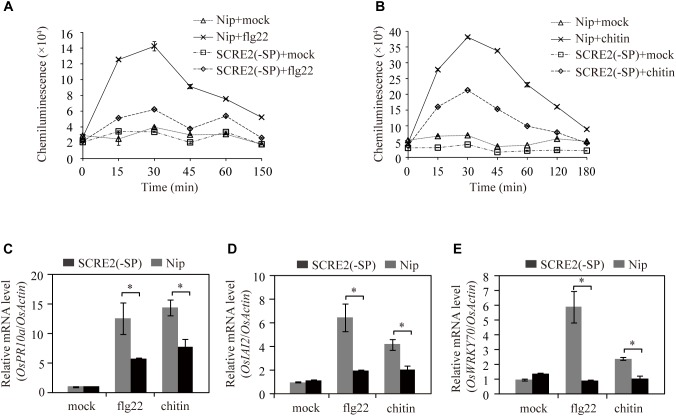
Heterologous expression of SCRE2 in transgenic rice cells inhibits rice immunity. **(A,B)** ROS burst induced by flg22 **(A)** and chitin **(B)** was significantly suppressed in *SCRE2(-SP)* transgenic cell cultures compared with that in the wild-type (Nip) cells. ROS generation induced by flg22 and chitin was detected by luminol-based chemiluminescence assays. **(C–E)** Expression of the defense genes *OsPR10a* **(C)**, *OsIAI2* **(D)**, and *OsWRKY70* **(E)** triggered by flg22 and chitin was significantly inhibited in *SCRE2(-SP)* transgenic cell cultures compared with that in the wild-type cells. The *OsActin* gene was used as an internal control. Means ± standard errors are shown. Asterisks (^∗^) indicate significant difference in the expression levels of defense marker genes between the wild-type and *SCRE2(-SP)* transgenic cell lines (*P* < 0.05). *SCRE2(-SP)*: the *SCRE2* gene lacking DNA sequences encoding the signal peptide.

To investigate whether SCRE2 has an ability to suppress plant immunity in host rice, we generated transgenic rice cell lines with SCRE2 expression driven by the 35S promoter. Different types of PAMPs including flg22 and chitin triggered a strong and rapid generation of ROS in rice cell cultures developed from the cultivar Nipponbare. In contrast, the oxidative burst triggered by flg22 and chitin was significantly suppressed in the SCRE2-expressing transgenic cells (Figure 5A,B). Furthermore, expression of defense marker genes, such as *OsPR10a*, *OsIAI2*, and *OsWRKY70*, was dramatically induced by flg22 and chitin in the wild-type cell suspensions compared with mock treatments. However, PAMP-induced expression of these defense genes was significantly suppressed in the *SCRE2* transgenic cell line (Figure 5C–E). These results indicate that heterologous expression of SCRE2 in host rice inhibits PAMP-triggered immunity.

### *SCRE2* Is Required for *U. virens* Virulence to Rice

**FIGURE 6:**
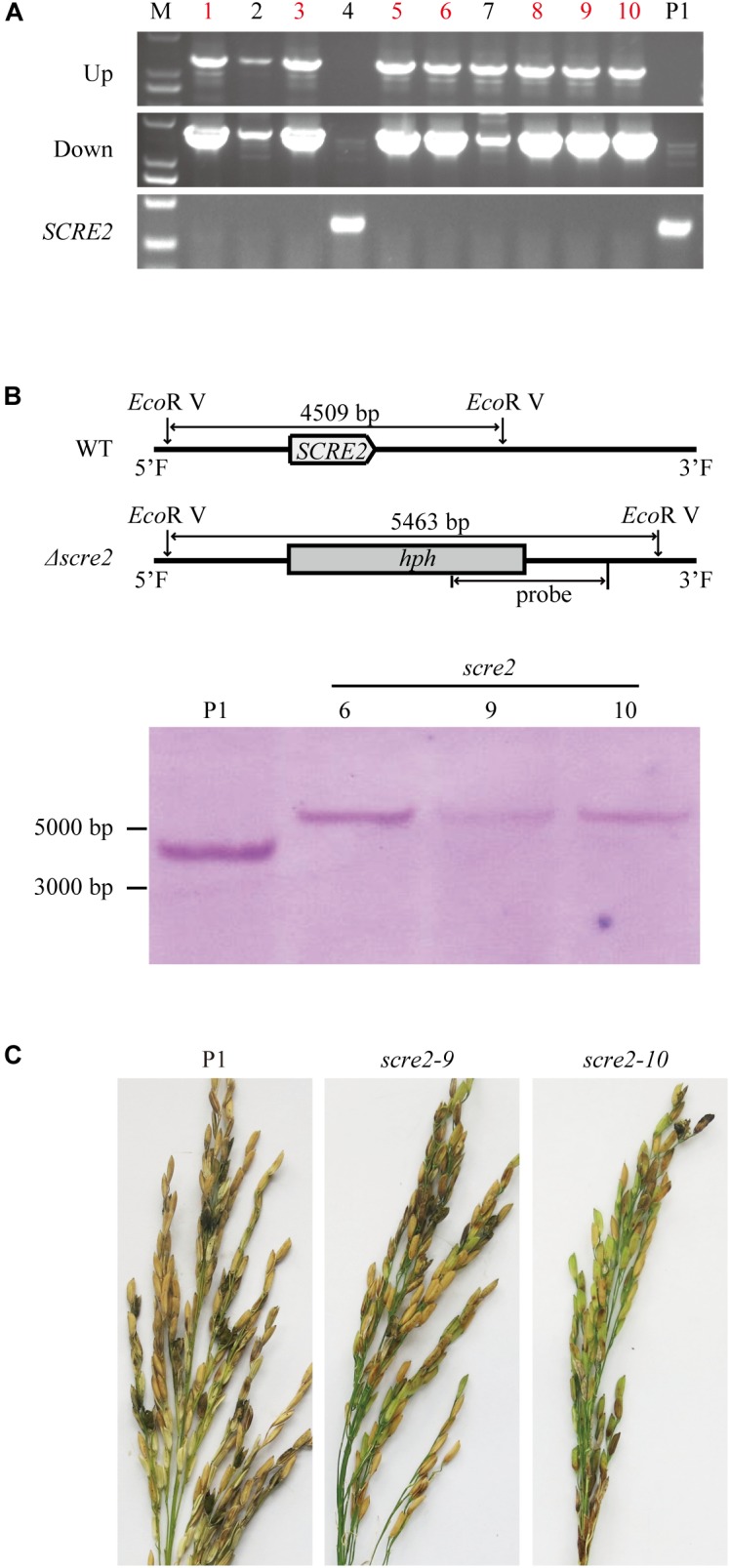
*SCRE2* is required for full virulence of *U. virens* to rice. **(A)** Screening of the *SCRE2* knockout mutants by diagnostic PCR. The upstream and downstream flanking sequences of hygromycin phosphotransferase (*hph*) gene and the *SCRE2* gene were amplified by PCR with designed primers, respectively (Supplementary Figure S6 and Supplementary Table S2). The absence of the PCR product of *SCRE2* and successful amplification of upstream and downstream jointing sequences of *hph* and *SCRE2* indicate that *SCRE2* is successfully replaced by *hph* gene via homologous recombination. The wild-type P1 strain was used as a negative control. M, molecular marker. **(B)** validation of the *SCRE2* knockout mutants via Southern blot analysis. Genomic DNA was isolated from the wild-type P1, *scre2*-6, *scre2*-9, and *scre2*-10 mutants and was then digested with *Eco*R V. After being separated on agarose gels, DNA was blotted onto nylon membrane. The digested DNA was then hybridized with isotope-labeled *SCRE2* probe. **(C)** Disease symptoms on rice panicles were observed about 1 month after inoculation with the *U. virens* wild-type and mutant strains. The wild-type P1 and mutant strains including *scre2*-9 and *scre2*-10 were inject-inoculated into rice panicles of LYP9 at 5∼7 days before rice heading.

To further investigate the role of *SCRE2* in *U. virens* virulence, gene-replacement mutants of *SCRE2* were generated via the CRISPR/Cas9 system. Nine out of ten independent *SCRE2*-replacement mutant candidates were verified by diagnostic PCR (Figure 6A and Supplementary Figure S6). Among them, three mutants including *scre2*-6, *scre2*-9, and *scre2*-10 were further confirmed via Southern blot analyses (Figure 6B). The wild-type, *scre2-9* and *scre2-10* strains were then inoculated into young panicles of the susceptible rice cultivar LYP9, RFS incidence and disease symptoms were investigated about 4 weeks after inoculation. The sporeballs formed in each inoculated panicle and the disease incidence rate were recorded and summarized in Table 1. Remarkably, the panicle incidence rate (%) caused by *scre2-9* and *scre2-10* was significantly lower than that caused by the P1 strain (Table 1). Similarly, the diseased grains per inoculated panicle caused by the mutants were also less than those caused by P1 in three independent experiments (Table 1 and Figure 6C). Therefore, *SCRE2* knock-out significantly attenuated *U. virens* virulence, indicating that SCRE2 contributes to full virulence of *U. virens.*

**Table 1:** The disease index in rice panicles after *U. virens* artificial inoculation.

	Strains	Panicle incidence rate (%)	Diseased grains/inoculated panicle	*P-*value^∗^
1st repeat	P1	100	10.9 ± 1.1	
	*scre2-9*	90	6.7 ± 1.0	0.011462
	*scre2-10*	60	1.5 ± 0.5	6.38E-08
2nd repeat	P1	90	12.1 ± 1.6	
	*scre2-9*	10	0.2 ± 0.2	3.85E-07
	*scre2-10*	0	0.0 ± 0.0	3.72E-07
3rd repeat	P1	65	6.0 ± 1.2	
	*scre2-9*	30	1.8 ± 1.0	0.012825
	*scre2-10*	30	2.0 ± 1.1	0.020498


**^∗^Statistical significance between the wild-type P1 and mutant strains was determined by Student’s t-test using Microsoft Excel. At least 10 panicles were inoculated for each strain. The diseased panicles and false smut balls formed on the inoculated panicles were counted about 1 month after inoculation. Data are means ± standard errors.**

## Discussion

*U. virens* has become one of the most important rice fungal pathogens in the past decade. *U. virens* genome encodes more than 600 secreted proteins, among which are 193 candidate effectors (Zhang et al., 2014). The majority of effectors in phytopathogenic microbes generally function as virulence factors, which target different essential components in the host defense signaling and suppress plant immunity (Giraldo and Valent, 2013; Lo Presti et al., 2015). Our previous studies indicate that many putative *U. virens* effectors might be involved in suppressing immunity-associated responses (Zhang et al., 2014). However, little is known about virulence functions of individual *U. virens* effectors. In this study, we identified and characterized a novel effector SCRE2 in *U. virens*. The effector suppresses PAMP-triggered immunity in host rice cell culture and hypersensitive responses triggered by different elicitors in the non-host *N. benthamiana*. It was further demonstrated that *SCRE2* is required for full virulence in *U. virens*, indicating that SCRE2 is an important virulence factor.

The small cysteine-rich protein SCRE2 carries a predicted N-terminal SP for secretion (Supplementary Figure S1). The prediction was supported by the yeast secretion assay, in which the putative SP of SCRE2 was functional to guide the secretion of the truncated invertase (Figure 1A). SCRE2 secretion was further confirmed by expressing SCRE2-mCherry in *N. benthamiana* leaves. Subcellular localization after plasmolysis showed that red fluorescence from SCRE2-mCherry was observed in the periplasmic spaces. The predicted SP is essential for SCRE2 localization in the extracellular matrix in *N. benthamiana* (Figure 2B). The expression pattern of *SCRE2* during infection also supports that the gene encodes an effector (Figure 1C). However, the actual action site of the effector needs to be further elucidated since SCRE2 localization assays were mainly performed in the heterologous system. Confocal microscopy showed that SCRE2-GFP was localized to cytoplasm and nucleus when SCRE2-GFP was transiently expressed in rice protoplasts and in *N. benthamiana* (Figure 2 and Supplementary Figure S4). Here, the artifact of protein diffusion into other organelles cannot be ruled out since free GFP was also localized to multiple cellular compartments and SCRE2-GFP was overexpressed. In addition, the facts that red fluorescence from SCRE2-mCherry is clearly visible in the apoplast of plasmolyzed *N. benthamiana* cells and that SCRE2 is a cysteine-rich protein may indicate its possible action and role in the apoplast, in analogy with *Ustilago maydis* Pit2 (Mueller et al., 2013). By contrast, live-cell imaging revealed that ectopically expressed SCRE2 in *M. oryzae* was translocated into plant cells during infection (Figure 1). Red fluorescence from mCherry-labeled SCRE2 was clearly visible in the nuclei of *M. oryzae*-infected barley cells through microscopy, indicating that SCRE2-mCherry-NLS is indeed secreted and taken up into barley cells (Figure 1B). Therefore, the actual localization of the effector will be only clarified in the *U. virens*/rice pathosystem. It is well known that the effectors from bacterial pathogens are delivered into host cells by the type III secretion system (Collmer et al., 2002). Intracellular effectors of plant filamentous pathogens are presumably secreted into extracellular spaces under the guide of N-terminal SPs before being transited into the host cells (Dou and Zhou, 2012). In oomycetes, the RxLR-dEER motif acts as a translocation signal for many intracellular effectors (Dou et al., 2008b), while some CRN effectors utilize a FLAK motif for translocation (Schornack et al., 2010). The cytoplasmic effectors in *M. oryzae*, such as AvrPiz-t and PWL2, are accumulated in the biotrophic interfacial complex (BIC) during infection and are then translocated into plant cells (Khang et al., 2010; Park et al., 2012). However, it is unknown yet whether cytoplasmic effectors in many fungal pathogens including *U. virens* are translocated through a special structure or under the guide of unidentified motifs.

The effector repertoir even in closely-related filamentous pathogens is largely different and the effectors generally lack sequence similarity and conserved motifs/domains probably due to positive selection and the action of transposons (Sperschneider et al., 2015). The putative effectors in *U. virens* share a very low amino-acid sequence identity with those in *Metarhizium anisopliae*, one of the evolutionarily closest species of *U. virens* among the species with available genome sequences so far (Zhang et al., 2014). Consistently, neither SCRE2 homologs nor conserved domains were identified in other species through BLAST and Pfam searches. By contrast, we revealed that the coding sequences of *SCRE2* are identical among 33 *U. virens* isolates collected from different Provinces in China and from Japan, India, and United States (Supplementary Figure S3 and Supplementary Table S1). Sequence conservation of SCRE2 implies its importance in *U. virens* virulence. Since pathogen effectors are often diversified under high-speed positive selection during the host-pathogen co-evolution, we hypothesize that SCRE2 has not been subject to intense positive selection or is not under surveillance by host R proteins.

In biotrophic and semi-biotrophic plant pathogens, many effector proteins have been demonstrated to inhibit plant cell death and thus promoting pathogen infection (Wang et al., 2011; Zhang et al., 2014; Chen et al., 2018). Avr1b and Avh331 from *P. sojae* inhibit cell death induced by the pro-apoptotic protein BAX and contribute positively to virulence, indicating that suppression of immunity-associated cell death by effectors is a feasible strategy for pathogen infection (Dou et al., 2008a). The effectors Avr3a and Pi17316 inhibiting cell death triggered by INF1, a PAMP recognized by a receptor-like protein (RLP) known as elicitin-response receptor (ELR) in potato, are required for full virulence of *P. infestans* (Bos et al., 2006; Bos et al., 2010; Du et al., 2015; Murphy et al., 2018). In this study, we demonstrated that SCRE2 suppressed BAX- and INF1-induced cell death in *N. benthamiana* (Figure 3, 4). Moreover, SCRE2 has been previously shown to inhibit non-host cell death induced by *B. glumae* in *N. benthamiana* (Zhang et al., 2014). More convincingly, we demonstrated that ectopic expression of SCRE2 in host rice cells significantly suppressed PAMP-triggered defense gene expression and oxidative burst (Figure 5). The results indicate that SCRE2 functions as a suppressor of plant immunity. Interestingly, SCRE2^68-85^, among a series of truncated SCRE2 proteins, was identified as a short functional region that retains the ability to suppress BAX-induced cell death and ROS generation (Figure 4). Here, it is necessary to mention that multiple attempts to detect via western blot analysis the expression of the small peptides in *N. benthamiana* failed, most likely due to instability and/or low levels of expression (Supplementary Figure S5). It is well known that a number of small regions in proteinaceous PAMPs can trigger plant immunity (Boutrot and Zipfel, 2017). However, to our knowledge, few studies have been reported on small regions in pathogen effectors that retain to have the immunosuppressive ability. Therefore, it is of interest to investigate molecular mechanisms on how SCRE2 and its small peptide region functions to inhibit plant immunity.

Many putative effector genes including *SCRE2* were significantly induced in rice spikelets during early stages of *U. virens* infection, suggesting that these genes probably contribute to virulence of the fungal pathogen (Figure 1C; Zhang et al., 2014). Gene knock-down and knock-out are valuable tools to use for determining the role of fungal effectors in pathogen virulence. The *Ustilago maydis* mutants with deletion of the effector genes *Pit2* and *Pep1* induce strong defense responses, which prevent fungal colonization and proliferation (Mueller et al., 2013). *Fusarium graminearum*, when deleting the *FGL1* effector gene, has a restricted ability to infect wheat spikelets (Blumke et al., 2014). Similarly, the *scre2* deletion mutant in *U. virens* exhibited an attenuated virulence to rice after artificial inoculation into rice panicles (Figure 6 and Table 1). Through screening a T-DNA insertion mutant library, some virulence factors in *U. virens* have been recently demonstrated to regulate virulence and pathogenicity (Yu et al., 2015). This study illustrates that an individual effector in *U. virens* contributes to full virulence to rice.

In summary, we identified and characterized a novel small cysteine-rich effector SCRE2 in *U. virens* that suppresses plant immunity in the host and non-host plants and therefore promotes pathogen virulence. However, the precise molecular mechanism how SCRE2 functions in shaping the rice – *U. virens* interaction is to be further elucidated.

## Materials and Methods

### Microbial Strains and Growth Conditions

The *U. virens* isolates UV-8b and P1 were cultured in PSA medium (boiled extracts from 200 g fresh potato, 20 g sucrose, and 14 g agar per liter). *N. benthamiana* plants were grown in growth chambers under a 25°C, 14 h day and 23°C, 10 h night cycle. *A. tumefaciens* GV3101 and EHA105 strains were cultured in Luria Bertani broth (0.5% yeast extract, 1% tryptone, 1% NaCl). The yeast strain YTK12 was cultured in YPDA medium (1% yeast extract, 2% peptone, 2% glucose, 0.003% adenine hemisulfate, 2% agar). *M. oryzae* P131 was cultured in oatmeal-tomato agar (OTA) medium (150 mL of tomato juice and 850 mL of boiled extracts from 30 g oat, pH 7.0). Antibiotics were used at the following concentrations (μg mL^-1^): ampicillin, 50; kanamycin, 50; rifampin, 25. All experiments in this study have been repeated independently at least three times with similar results unless noted.

### RNA Isolation and Plasmid Construction

Total RNAs were extracted from *U. virens* mycelia, rice cell cultures and inoculated rice panicles using an Ultrapure RNA isolation kit according to the manufacturer’s instructions (CWBio, Beijing) and were quantified via NanoDrop 2000 (Thermo Fisher Scientific, United States). Complementary DNA was synthesized using 2 μg of total RNAs by Superscript III reverse transcriptase (Invitrogen, Carlsbad, CA, United States). The coding sequences of *SCRE2* and its truncated variants were amplified from cDNAs using fast *Pfu* polymerase (TransGen) with the respective primer sets in Supplementary Table S2. PCR products were subcloned into pGR107 (Jones et al., 1999) after digestion with *Xma* I and *Sal* I or *Xho* I. All constructs were confirmed by sequencing.

### Transient Expression in *N. benthamiana*

The constructed plasmids were transformed into *Agrobacterium* GV3101 through the freeze-thaw method (Holsters et al., 1978). For transient expression in *N. benthamiana*, overnight-cultured *Agrobacterium* strains were collected and washed twice with distilled dH_2_O, and were then resuspended in 10 mM MgCl_2_ with 150 μM acetosyringone and 10 mM MES, pH 5.7 to suitable concentrations. After incubation for 3 h or more at room temperature, *Agrobacterium* cells carrying the BAX (OD_600_ ∼ 1.0) and SCRE2 (OD_600_ ∼ 0.6) gene constructs were equally mixed and infiltrated into 4 weeks old *N. benthamiana* plant leaves with needleless syringes. Cell death symptoms were recorded at 3–4 days after infiltration.

### Ion Leakage in Leaf Discs of *N. benthamiana*

In order to quantify BAX-triggered cell death in *N. benthamiana* leaves, ion leakage was measured in the agro-infiltrated leaves as described previously (Mittler et al., 1999; Fang et al., 2016).

### Yeast Secretion Assay

The yeast secretion assay was performed as described previously (Jacobs et al., 1997; Fang et al., 2016). The vector pSUC2T7M13ORI (pSUC2) carrying a truncated invertase gene (*SUC2*) lacking its own SP coding sequence and the start codon was used in this assay (Oh et al., 2009). The predicted SP coding sequence of *SCRE2* was amplified using the primers in Supplementary Table S2 and subcloned into pSUC2. The constructed plasmid (0.5 μg) was transformed into the invertase-deficient yeast strain YTK12 using the Frozen-EZ yeast transformation II kit (Zymo Research). The functionality of predicted SP was judged by the growth of YTK12 on YPRAA medium (1% yeast extract, 2% peptone, 2% raffinose, and antimycin A at 2 μg/L).

### Subcellular Localization of SCRE2 and SCRE2 Without Signal Peptide

The coding sequences of *SCRE2* and *SCRE2-SP* (without SP) were amplified and ligated into the pGD-GFP and pGD-mCherry plasmids (Goodin et al., 2010) after digestion with *Xho* I and *Bam*H I, and pUC19-GFP-3 × FLAG after digestion with *Xho* I and *BstB* I, respectively (Supplementary Table S2). The pGD constructs were transformed into *Agrobacterium* strain GV3101 and agro-infiltrated into *N. benthamiana* leaves. The leaves were stained and observed at 2 days after infiltration. FM4-64 [N-(3-triethylammom iumpropyl)-4-(p-diethylam inophenylhexatrienyl)] and DAPI (4′, 6-diamidino-2-phenylindole) staining was performed as described previously (Ueda et al., 2001; Kim et al., 2016; Liu et al., 2017). The infiltrated leaves were detached and submerged in 8.2 μM of FM4-64 solution for 15 min. Alternatively, the detached leaves were immersed into DAPI (5 μg/mL) solution with 0.01% Silwet L-77 for 5 min. The stained leaves were rinsed with distilled water for three times and observed immediately. For subcellular localization in rice cells, the protoplasts were isolated from rice seedlings and transformed with pUC19-*GFP-3 × FLAG* constructs as described previously (Fang et al., 2016). To detect apoplastic localization of SCRE2, SCRE2-mCherry, SCRE(-SP)-mCherry, and mCherry were transiently expressed in *N. benthamiana* leaves. The leaves were detached and treated with 1 M NaCl to induce plasmolysis. GFP, FM4-64, mCherry and DAPI signals were detected in the lower epidermal cells by a confocal scanning laser microscope (OLYMPUS) with standard filter sets.

### Development of *SCRE2(-SP)* Transgenic Cell Lines

The coding sequence of *SCRE2(-SP)* was amplified from cDNAs with specific primers (Supplementary Table S2). The amplified fragment was cloned into the pENTR TOPO entry vector (Invitrogen), and was then recombined into the binary plasmid pGWB11 (Nakagawa et al., 2007) via Gateway LR clonase II enzyme mix (Invitrogen) according to the manufacturer’s instructions. The pGWB11-*SCRE2(-SP)* construct was introduced into *Agrobacterium tumefaciens* EHA105 by the freeze-thaw method. The construct was then transformed into rice calli through *Agrobacterium tumefaciens*-mediated transformation as described previously (Li et al., 2015). Rice cell-suspension cultures from the wild type (Nip) and *SCRE2(-SP)* transgenic rice calli were developed as described previously (Li et al., 2015).

### Protein Extraction and Immunoblotting

*N. benthamiana* leaves were harvested at 36 h after agro-infiltration and were then ground in liquid nitrogen. Rice protoplasts were collected at 16 h after transfection. The powder or protoplasts were incubated with 1 × SDS-PAGE sample buffer and then boiled for 10 min. The protein samples were then separated on a 12% SDS-polyacrylamide gel and were electrophoretically blotted onto nitrocellulose membranes (Millipore). After being stained with 0.1% Ponceau S to visualize protein loading, the membranes were blocked overnight with 5% skimmed milk in TBS-T buffer (50 mM Tris-Cl, pH 7.5, 150 mM NaCl, 0.05% Tween 20) at 4°C. The membranes were incubated with anti-Flag, anti-GFP or anti-mCherry (1:5,000 dilution) for 1 h at room temperature. After washing with TBS-T buffer, the blots were incubated in anti-mouse or anti-rabbit HRP-conjugated secondary antibody (1:5,000 dilution in TBS-T) for 1 h at room temperature. Finally, the immunoblots, after rinsing thoroughly, were incubated with the eECL western substrate (CWBio) and were then exposed to X-films.

### ROS Assays

The generation of ROS was detected via 3, 3′-diaminobenzidine (DAB) staining (Thordal-Christensen et al., 1997; Liu et al., 2016). Briefly, *N. benthamiana* leaves were collected at 2, 3, and 4 days after co-infiltration with *Agrobacterium* cells carrying pGR107-*BAX* and pGR107-*GFP* or pGR107-*SCRE2^68-85^* and were then placed in the DAB solution (1 mg/mL, Sigma-Aldrich) for 12 h with shaking at 80 rpm. Subsequently, the leaves were cleared in 96% ethanol and imaged. The ROS burst in rice cell suspension cultures was detected by a luminol-dependent chemiluminescence assay as described previously with minor modifications (Lu et al., 2015). Briefly, cell cultures were washed three times and then pre-incubated in 3 mL of fresh media with shaking at 28°C for 24 h. The cultured cells were treated with 1 μM flg22, 20 μL of 100 mg/mL chitin or sterile dH_2_O (mock control) for different times. The supernatant (10 μL) collected at different time points was added into 1 mL of Co(II)-luminol mixed reagent. Chemiluminescence was measured immediately by the Infinite F200 (Tecan, Männedorf, Switzerland).

### Quantitative Real-Time RT-PCR

Gene expression was determined via quantitative real time RT-PCR by specific primers listed in Supplementary Table S2. RNA isolation and reverse transcription were performed as describe above. Quantitative RT-PCR was done with an ABI PRISM 7000 Sequence Detection System (Applied Biosystems, Foster City, CA, United States). The reactions were performed in a volume of 20 μL containing 0.5 μL cDNA, 10 μL SYBR^®^ premix Ex Taq (TaKaRa), and 0.2 μM of the forward and reverse primers with the following programs, an initial step for 30 s at 95°C, followed by 40 cycles of denaturation for 5 s at 95°C, annealing and elongation for 30 s at 60°C. A melting curve analysis was performed over a temperature range of 60–95°C, with stepwise 1°C increments in the temperature to verify specific amplification. The α*-tubulin* and *OsActin* genes were used as internal controls for *U. virens* and rice gene expression, respectively.

### The Translocation Assay

The coding sequence of *SCRE2* was amplified and then subcloned into the modified pKS vector (Park et al., 2012), by which a fusion protein with a nucleus localization sequence (NLS) and mCherry is expressed under the control of RP27 promoter (Bourett et al., 2002). The pKS-*RP27*::*SCRE2-mCherry-NLS* construct was transformed into the *M. oryzae* strain P131 via PEG-mediated transformation as described (Sweigard et al., 1992; Yang et al., 2010). The successful transformants were screened on potato-dextrose agar plates supplemented with 400 μg/mL neomycin (Amresco). The expression of SCRE2-mCherry-NLS in *M. oryzae* was confirmed by fluorescence microscopy Nikon Eclipse 90i. *M. oryzae* conidiation was induced as described previously (Peng and Shishiyama, 1988). For barley inoculation, the conidial suspension (1 × 10^5^ conidia/mL) in 0.025% Tween 20 was spotted onto the lower epidermis of detached young barley (*Hordeum vulgare*) leaves and then incubated in a moist, dark chamber at 28°C. Red fluorescence was observed at 30 hpi via fluorescence microscopy.

### Generation of the *SCRE2* Replacement Mutants

The *SCRE2* replacement mutants were created using the CRISPR/Cas9 system (Zheng et al., 2016; Liang et al., 2018). The specific sgRNA primers for targeting *SCRE2* were designed following the procedure on the website^1^. The synthesized primers were annealed and subcloned into pCAS9-tRp-gRNA (Liang et al., 2018; Supplementary Table S2). The upstream and downstream flanking sequences of *SCRE2* were amplified from genomic DNA with the primer pairs SCRE2-1F/SCRE2-2R and SCRE2-5F/SCRE2-6R, respectively (Supplementary Table S2). The amplified products were connected to the 5′- and 3′-termini of the *hptII* gene, which was amplified from pGKO2 with the primer set SCRE2-3F/SCRE2-4R, by fusion PCR, respectively (Supplementary Table S2). PEG-mediated *U. virens* transformation was performed as described (Zheng et al., 2016). Briefly, the collected *U. virens* conidia were cultured in 50 mL of PS medium at 28°C with shaking at 150 rpm for 18–20 h. The germinated hyphae were collected by filtering with three-ply lens paper and then rinsed with 1.2 M KCl twice. The hyphae were incubated in Lysis solution (25 g/L Driselase, 10 g/L Lysing enzyme, 0.05 g/L lyticase, 1.2 M KCl) at 30°C with shaking at 90 rpm for 3 h. The released protoplasts were harvested through filtration with three-ply lens paper followed by centrifugation at 3,500 rpm for 10 min. The protoplasts were rinsed with STC solution (200 g/L sucrose, 0.05 M Tris-Cl, pH 8.0, 0.05 M CaCl_2_⋅2H_2_O) twice and was then adjusted to 1 × 10^8^ /mL in STC. The PCR product (5 μg) and the pCAS9-tRp-gRNA construct (5 μg) were mixed with 150 μL of *U. virens* protoplasts and were incubated for 20 min. PTC solution (40% PEG8000 in STC solution, 1 mL) was then added and incubated for another 20 min, followed by adding 5 mL of TB3 (3 g/L yeast extract, 3 g/L casamino acids, 20% sucrose). After incubating at 28°C with shaking at 90 rpm for 36 h, the protoplasts were mixed with 40 mL of TB3 bottom medium (10 g/L agar) and divided into four plates. These plates were cultured overnight at 28°C and then overlaid with 12.5 mL of TB3 top medium (10 g/L agar with 200 μg/mL hygromycin B) for transformant selection.

### Virulence Assays of *U. virens* to Rice

Virulence assays of *U. virens* to rice were performed as described previously (Wang et al., 2008; Han et al., 2015). Briefly, the *U. virens* strains were cultured in PS medium at 28°C with shaking at 180 rpm for 6 days. After smashing with blender, the mixture of hyphae and spores were diluted to 1 × 10^6^ spores/mL in PS medium and were then injected into rice panicles of LYP9 at 5–7 days before rice heading. At least 10 panicles were inoculated for each strain. False smut balls formed on rice panicles were counted 4 weeks after inoculation.

## Author Contributions

WS and AF designed and conceived the project. AF, HG, NZ, XZ, SQ, YL, SZ, and FC performed the experiments and analyzed the data. WS and AF wrote the manuscript with contributions from all the authors. All authors read and approved the final version of the manuscript for publication.

## Conflict of Interest Statement

The authors declare that the research was conducted in the absence of any commercial or financial relationships that could be construed as a potential conflict of interest.

## Footnotes

1 http://www.rgenome.net/

## Supplementary Material

Figure S1 Amino acid sequences derived from the open reading frame of *SCRE2*. *SCRE2* encodes a protein with 130 amino acid residues. Underlined is the predicted SP of SCRE2. The 68–85 amino acid residues as a functional domain suppressing BAX-triggered plant cell death are highlighted in red and bold. The Cys residues in SCRE2 are indicated by asterisks.

Figure S2 SCRE2-mCherry-NLS was ectopically expressed in *Magnaporthe oryzae*. The gene construct *SCRE2-mCherry-NLS* was transformed into *M. oryzae* P131 via PEG-mediated transformation. Red fluorescence observed via fluorescence microscopy indicates the expression of SCRE2-mCherry-NLS in *M. oryzae* conidia. NLS, nuclear localization signal.

Figure S3 Sequence alignment of the *SCRE2* genes from 33 different *U. virens* isolates collected from Japan, India, America, and different regions in China. The *SCRE2* gene sequences of UV_8b, Japan and Indian isolates were downloaded from PubMed, while other *SCRE2* sequences were determined by sequencing PCR products.

Figure S4 Expression and subcellular localization of fluorescently tagged SCRE2 in *N. benthamiana* and in rice protoplasts. **(A)** SCRE2-GFP was localized to the nuclei, cytoplasm and plasma membrane in rice protoplasts. Green fluorescence was observed in rice protoplasts transiently expressing GFP, SCRE2-GFP and SCRE2(-SP)-GFP via laser scanning confocal microscopy. GFP panels: green fluorescence; mCherry panels: red fluorescence was observed after mCherry-NLS was expressed in rice protoplasts to indicate nuclei; merged panels: overlay images of green and red fluorescence signals demonstrated that SCRE2-GFP and SCRE2(-SP)-GFP were also localized to the nuclei. Scale bar, 5 μM. NLS, nuclear localization signal. **(B)** SCRE2-GFP and SCRE2(-SP)-GFP were stably expressed in *N. benthamiana.* **(C)** GFP, SCRE2-GFP, and SCRE2(-SP)-GFP were stably expressed in rice protoplasts. **(D)** mCherry, SCRE2-mCherry and SCRE2(-SP)-mCherry were expressed in *N. benthamiana*. SCRE2(-SP): SCRE2 without the signal peptide. In **(B–D)**, upper panels, Western blot analyses probed with anti-GFP (α-GFP) and anti-mCherry (α-mCherry) antibody. Protein markers were shown at the left in kDa. Lower panels, Ponceau S staining indicated an equal loading of total proteins.

Figure S5 The expression level of different truncated SCRE2 proteins in *N. benthamiana* detected by western blot analyses. The FLAG-tagged truncated proteins of SCRE2 were detected with an anti-FLAG (α-FLAG) antibody. Several small truncated proteins were not detectable probably because they are too small to be detected via western blot analyses. Lower panels, Ponceau S staining indicated an equal loading of total proteins.

Figure S6 Schematic illustration to generate *SCRE2* gene replacement via CRISPR/Cas9 and the strategy of primer design for diagnostic PCR. The gRNA target site for *SCRE2* knockout was shown. The *SCRE2* gene, upstream and downstream jointing sequences of *hph* and *SCRE2* were amplified using the primer sets P1(F/R), P2(F/R), and P3(F/R), respectively.

Table S1 Thirty-three *U. virens* isolates from United States, India, Japan, and different Provinces in China used for *SCRE2* gene sequence analysis.

Table S2 The primers used in this study.
