# Supplementary figures and images for: A Novel Effector Gene SCRE2 Contributes to Full Virulence of Ustilaginoidea virens to Rice
Source: Front Microbiol. 2019 Apr 24;10:845. doi: 10.3389/fmicb.2019.00845 (PMC6492501; doi:10.3389/fmicb.2019.00845)

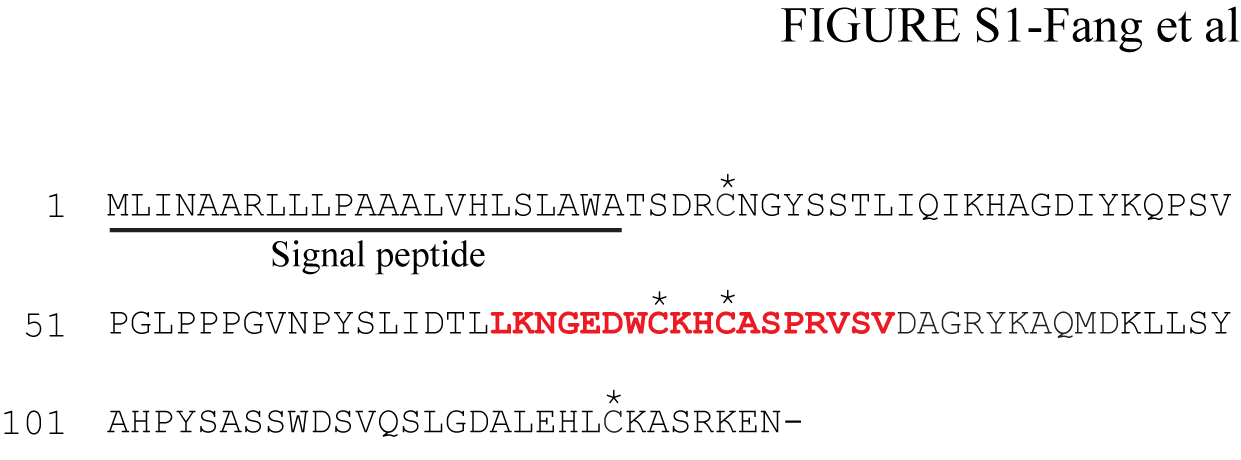

Supplement: Figure S1 — Amino acid sequences derived from the open reading frame of SCRE2. SCRE2 encodes a protein with 130 amino acid residues. Underlined is the predicted SP of SCRE2. The 68–85 amino acid residues as a functional domain suppressing BAX-triggered plant cell death are highlighted in red and bold. The Cys residues in SCRE2 are indicated by asterisks. [file Image_1.TIF]

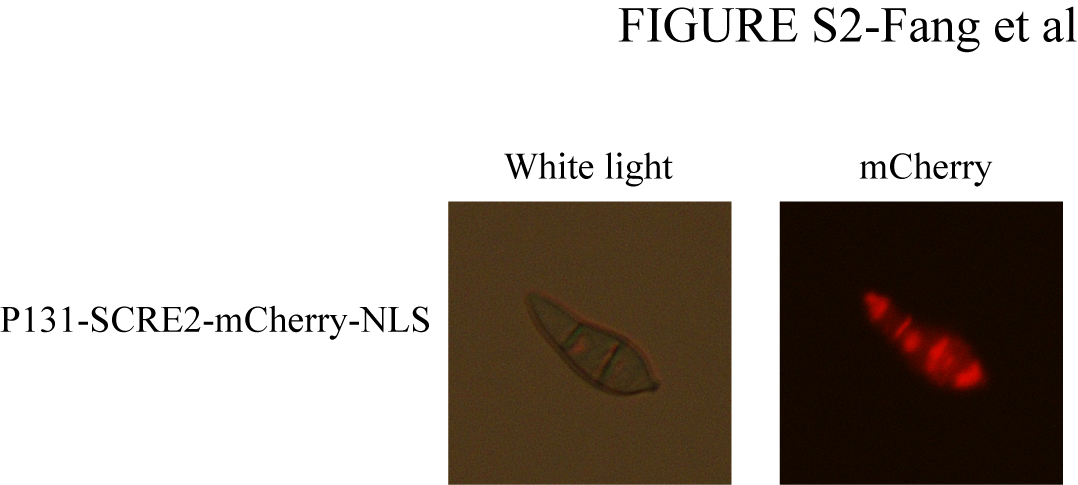

Supplement: Figure S2 — SCRE2-mCherry-NLS was ectopically expressed in Magnaporthe oryzae. The gene construct SCRE2-mCherry-NLS was transformed into M. oryzae P131 via PEG-mediated transformation. Red fluorescence observed via fluorescence microscopy indicates the expression of SCRE2-mCherry-NLS in M. oryzae conidia. NLS, nuclear localization signal. [file Image_2.TIF]

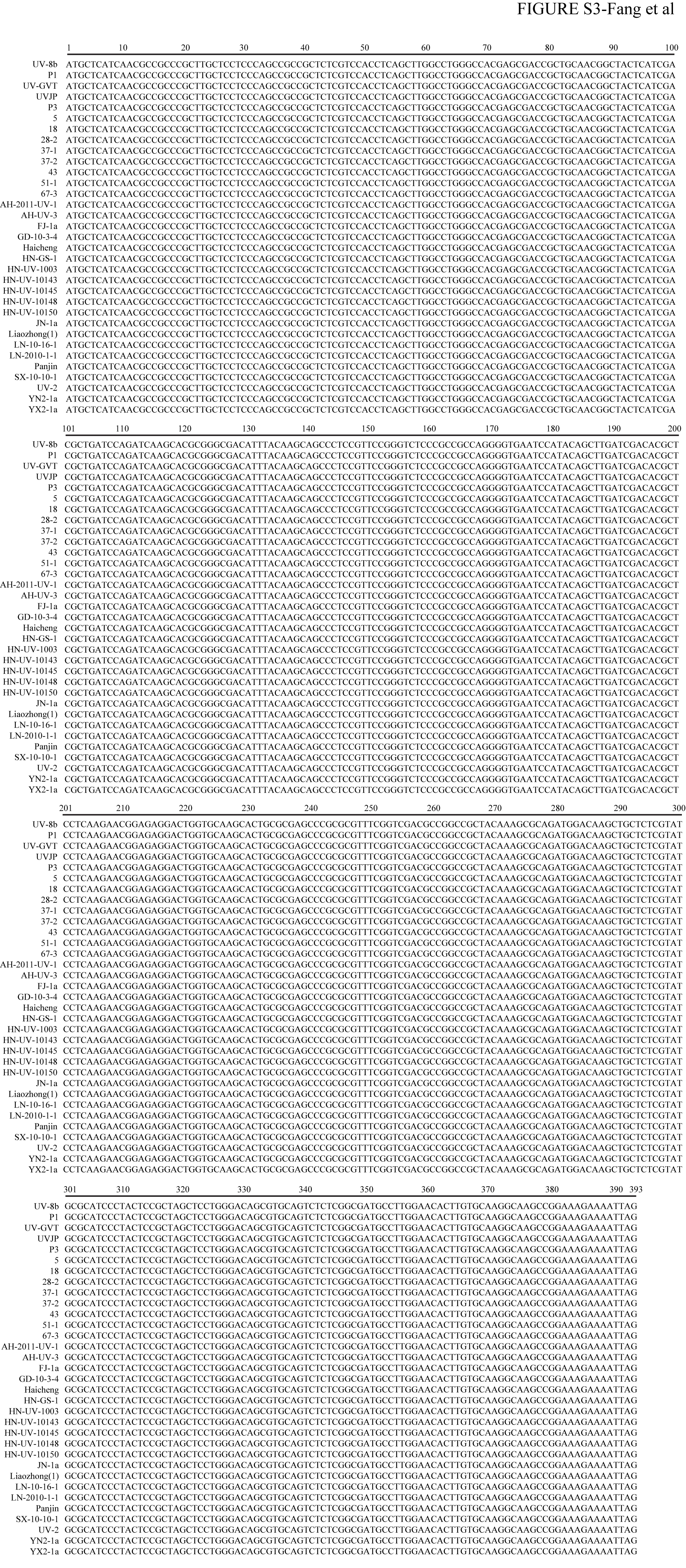

Supplement: Figure S3 — Sequence alignment of the SCRE2 genes from 33 different U. virens isolates collected from Japan, India, America, and different regions in China. The SCRE2 gene sequences of UV_8b, Japan and Indian isolates were downloaded from PubMed, while other SCRE2 sequences were determined by sequencing PCR products. [file Image_3.TIF]

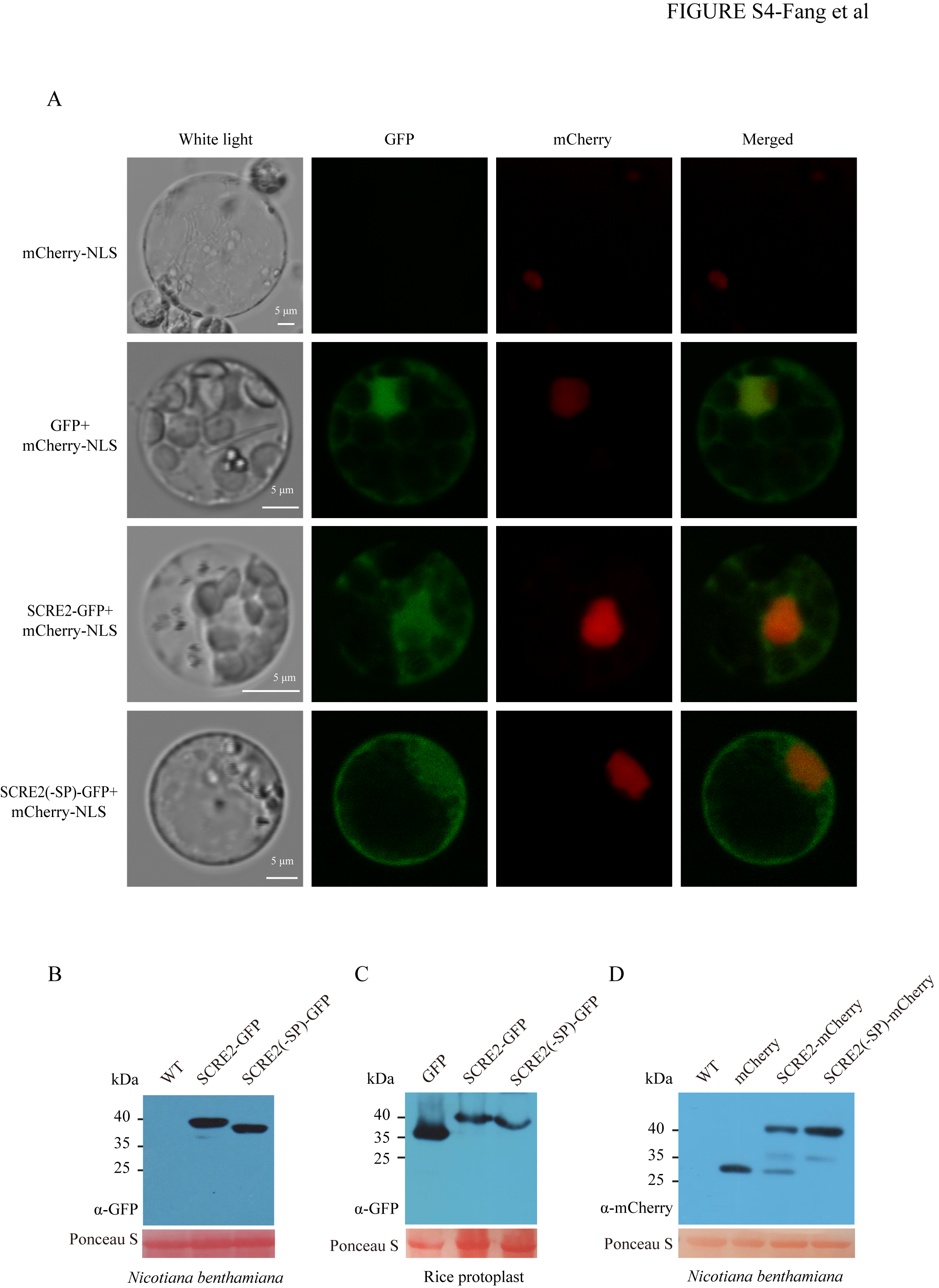

Supplement: Figure S4 — Expression and subcellular localization of fluorescently tagged SCRE2 in N. benthamiana and in rice protoplasts. (A) SCRE2-GFP was localized to the nuclei, cytoplasm and plasma membrane in rice protoplasts. Green fluorescence was observed in rice protoplasts transiently expressing GFP, SCRE2-GFP and SCRE2(-SP)-GFP via laser scanning confocal microscopy. GFP panels: green fluorescence; mCherry panels: red fluorescence was observed after mCherry-NLS was expressed in rice protoplasts to indicate nuclei; merged panels: overlay images of green and red fluorescence signals demonstrated that SCRE2-GFP and SCRE2(-SP)-GFP were also localized to the nuclei. Scale bar, 5 μM. NLS, nuclear localization signal. (B) SCRE2-GFP and SCRE2(-SP)-GFP were stably expressed in N. benthamiana. (C) GFP, SCRE2-GFP, and SCRE2(-SP)-GFP were stably expressed in rice protoplasts. (D) mCherry, SCRE2-mCherry and SCRE2(-SP)-mCherry were expressed in N. benthamiana. SCRE2(-SP): SCRE2 without the signal peptide. In (B–D), upper panels, Western blot analyses probed with anti-GFP (α-GFP) and anti-mCherry (α-mCherry) antibody. Protein markers were shown at the left in kDa. Lower panels, Ponceau S staining indicated an equal loading of total proteins. [file Image_4.TIF]

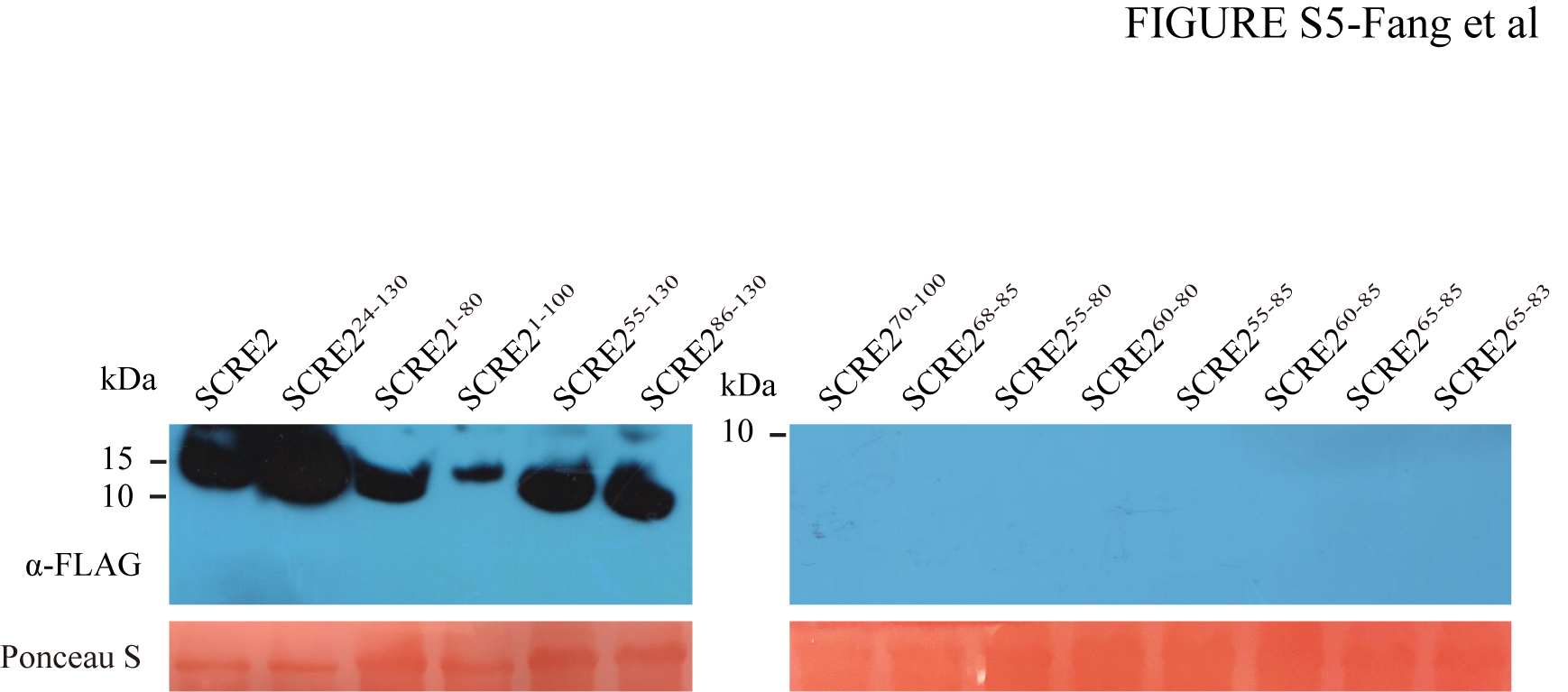

Supplement: Figure S5 — The expression level of different truncated SCRE2 proteins in N. benthamiana detected by western blot analyses. The FLAG-tagged truncated proteins of SCRE2 were detected with an anti-FLAG (α-FLAG) antibody. Several small truncated proteins were not detectable probably because they are too small to be detected via western blot analyses. Lower panels, Ponceau S staining indicated an equal loading of total proteins. [file Image_5.TIF]

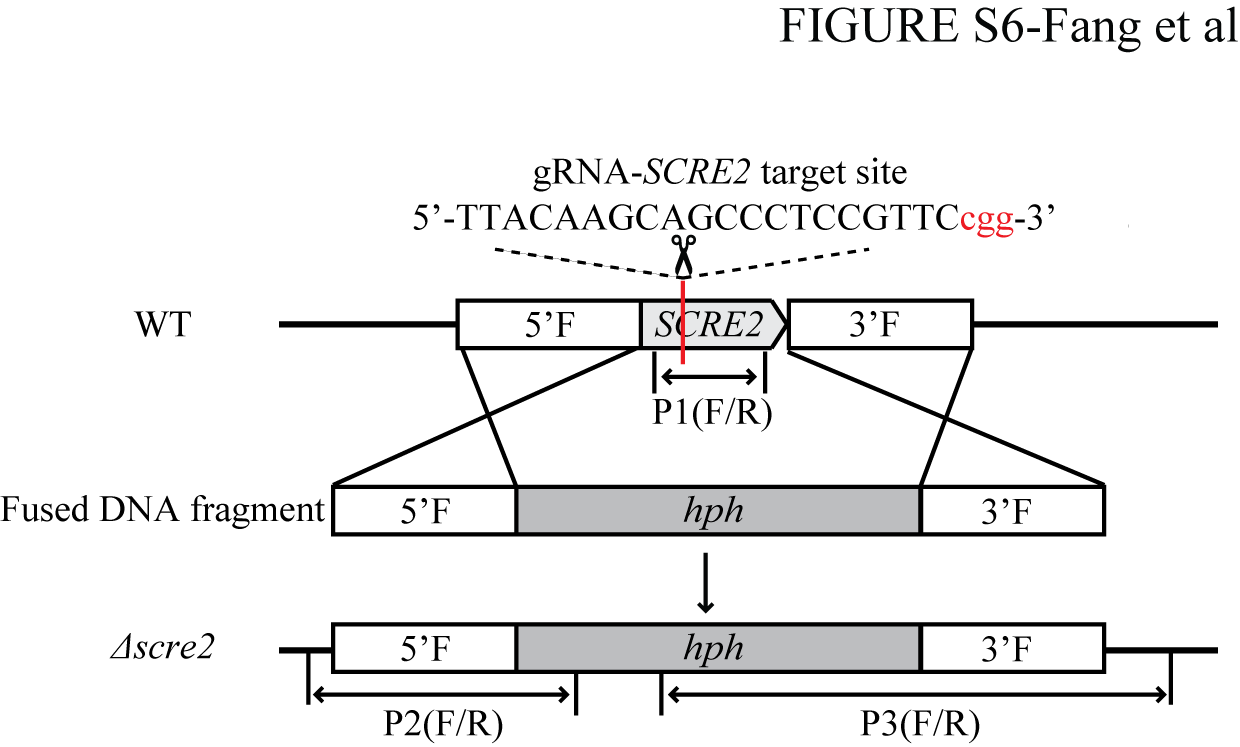

Supplement: Figure S6 — Schematic illustration to generate SCRE2 gene replacement via CRISPR/Cas9 and the strategy of primer design for diagnostic PCR. The gRNA target site for SCRE2 knockout was shown. The SCRE2 gene, upstream and downstream jointing sequences of hph and SCRE2 were amplified using the primer sets P1(F/R), P2(F/R), and P3(F/R), respectively. [file Image_6.TIF]
