# Supplementary material for: A Novel Effector Gene SCRE2 Contributes to Full Virulence of Ustilaginoidea virens to Rice
Source: Front Microbiol. 2019 Apr 24;10:845. doi: 10.3389/fmicb.2019.00845 (PMC6492501; doi:10.3389/fmicb.2019.00845)
Supplement: Table S1 — Thirty-three U. virens isolates from United States, India, Japan, and different Provinces in China used for SCRE2 gene sequence analysis. [file Table_1.DOCX]

**Table S1. Thirty-three *U. virens* isolates from USA, India, Japan and different Provinces in China used for *SCRE2* gene sequence analysis**

| Name | Rice cultivar | origin region |
| --- | --- | --- |
| UV-8b | Zhonghua11 | Wuhan, Hubei Province |
| P1 | Unknown | USA |
| UV-GVT | Unknown | India |
| UVJP | Unknown | Japan |
| P3 | Unknown | Unknown |
| 5 | Zhongyu16 | Xuzhou City, Jiangsu Province |
| 18 | Zhongyu27 | Xuzhou City, Jiangsu Province |
| 28-2 | W1160 | Suzhou City, Jiangsu Province |
| 37-1 | Unknown | Nanjing, Jiangsu Province |
| 37-2 | 80024 | Lishui County, Nanjing , Jiangsu Province |
| 43 | F9840 | Nanjing , Jiangsu Province |
| 51-1 | SF250 | Yancheng, Jiangsu Province |
| 67-3 | Changyou3 | Nantong, Jiangsu Province |
| AH-2011-UV-1 | Unknown | Anhui Province |
| AH-UV-3 | Guoxiang8 | Anhui Province |
| FJ-1a | Chuanyou673 | Nanjing County, Zhangzhou, Fujian Province |
| GD-10-3-4 | Unknown | Guangdong Province |
| Haicheng | Unknown | Haicheng, Liaoning Province |
| HN-GS-1 | Unknown | Luoshan County, Xinyang, Henan Province |
| HN-UV-1003 | TJ-1 | Taojiang County, Yiyang, Hunan Province |
| HN-UV-10143 | Jinyou287 | Hanshou County, Changde, Hunan Province |
| HN-UV-10145 | Fengyuanyou299 | Changde, Hunan Province |
| HN-UV-10148 | Jinmeiyou148 | Xiangtan，Hunan Province |
| HN-UV-10150 | 167AX432 | Anren County, Chenzhou, Hunan Province |
| JN-1a | Jinan-13 | Licheng District, Jinan, Shandong Province |
| Liaozhong(1) | Unknown | Liaozhong, Liaoning Province |
| LN-10-16-1 | Yuanshidashui | Donggang, Liaoning Province |
| LN-2010-1-1 | Unknown | Donggang, Liaoning Province |
| Panjin | Unknown | Panjin, Liaoning Province |
| SX-10-10-1 | Xianyou series | Nanzheng County, Hanzhong, Shanxi Province |
| UV-2 | Liangyoupeijiu | Nanjing, Jiangsu Province |
| YN2-1a | Baipinuo | Yunnan Province |
| YX2-1a | Aoyou938 | Yangxin County, Huangshi, Hubei Province |

**Table S2. The primers used in this study**

| Gene | Primer |
| --- | --- |
| **pSUC2 construction** | |
| SCRE2-*Eco*R I-F | at gaattc ATG CTC ATC AAC GCC GCC CGC T |
| SCRE2-*Xho* I-R | at ctcgag CGT CGA TGA GTA GCC GTT GCA G |
| **qRT-PCR primers** | |
| SCRE2-qRT-F | ATC CAG ATC AAG CAC GCG |
| SCRE2-qRT-R | CCA GTC CTC TCC GTT CTT G |
| *OsPR10a*-qRT-F | GAC ATC GTG GAT GGC TAC TAT GG |
| *OsPR10a*-qRT-R | TCA CTC ACT CTA GGT GGG ATA TAC |
| *OsIAI2*-qRT-F | AAA AGA AAA TGC CGT GGG TG |
| *OsIAI2*-qRT-R | AGA CAG GGT CGC ATT TCT TG |
| *OsWRKY70*-qRT-F | GTT TCA TTT GTT TCG GAG GCC |
| *OsWRKY70*-qRT-R | TTC TCC CTA TAC GCC CTC TGT G |
| *OsActin-*qRT-F | TCC ATC TTG GCA TCT CTC AG |
| *OsActin-*qRT-R | GTA CCC GCA TCA GGC ATC TG |
| *ɑ-tubulin-* qRT-F | AGG TTG CGT TGA AGG AGG TT |
| *ɑ-tubulin-* qRT-F | GAG GTG GAG TTG CCG ATA AA |
| **pGD-*GFP* construction** | |
| SCRE2-*Xho* I(CT)-F | att ctcgag ct ATG CTC ATC AAC GCC GCC CGC T |
| SCRE2(-SP)-*Xho* I(CT)-F | att ctcgag ct ATG ACG AGC GAC CGC TGC AAC GG |
| SCRE2-*Bam*H I-R | att ggatcc ATT TTC TTT CCG GCT TGC CTT GCA |
| **pUC19-*GFP-3×FLAG* construction** | |
| SCRE2-*Kpn* I-F | att ggtacc ATG CTC ATC AAC GCC GCC CGC T |
| SCRE2(-SP)-*Kpn* I-F | att ggtacc ATG ACG AGC GAC CGC TGC AAC GG |
| SCRE2-*Xho* I-R | att ctcgag ATT TTC TTT CCG GCT TGC CTT GCA |
| **pGD-*mCherry* construction** | |
| SCRE2-*Xho* I(CT)-F | att ctcgag ct ATG CTC ATC AAC GCC GCC CGC T |
| SCRE2(-SP)-*Xho* I(CT)-F | att ctcgag ct ATG ACG AGC GAC CGC TGC AAC GG |
| SCRE2-*Hin*d III-R | att aagctt g ATT TTC TTT CCG GCT TGC CTT GCA |
| **pGWB11-*SCRE2(-SP)* construction** | |
| SCRE2-F | CACC ATG ACG AGC GAC CGC TGC AAC GG |
| SCRE2-R | ATT TTC TTT CCG GCT TGC CTT GCA |
| **Sequence analysis of *SCRE2*** | |
| SCRE2-CA-F | TCC CCT TGG CTC ATT CTC CCT G |
| SCRE2-CA-R | TGG GCA CCC GTC TGC ATA GC |
| **PCR verification for *Δscre2*** | |
| P1-F | ATG CTC ATC AAC GCC GCC CG |
| P1-R | CTA ATT TTC TTT CCG GCT TGC |
| P2-F | AAT GGC GCT CCC AGA TGA GC |
| P2-R | TCT GGA CCG ATG GCT GTG TA |
| P3-F | GTT ATC GTG CAC CAA GCA GCA G |
| P3-R | GCT CAT CTC AAC AGC CTG CT |
| **Southern blot verification for *Δscre2*** | |
| SCRE2-probe-F | GCT GTG GAA TGG ATG GCT TGC |
| SCRE2-probe-R | GCA GCT CGG ATG GAG AAG ATG |
| **pCAS9-tRp vector construction** | |
| SCRE2-gRNA | TTA CAA GCA GCC CTC CGT TC |
| CRISPR-SCRE2-F | acct TTA CAA GCA GCC CTC CGT TC |
| CRISPR-SCRE2-R | aaac GAA CGG AGG GCT GCT TGT AA |
| **Soeing-PCR Primers for *SCRE2* knockout** | |
| SCRE2-1F | TCTTCCACTACGTCCCTCCG |
| SCRE2-3F | AGG CAA CTG CTT CAA CCA GCG ATA ACT GGT TCC CGG TCG G |
| SCRE2-2R | CCG ACC GGG AAC CAG TTA TCG CTG GTT GAA GCA GTT GCC T |
| SCRE2-5F | CCG CCT GAC GAC TAA ACC AAA CGG CTT GTG CGG CTA TGC AGA |
| SCRE2-4R | TCT GCA TAG CCG CAC AAG CCG TTT GGT TTA GTC GTC AGG CGG |
| SCRE2-6R | CTC AAC GCC GGA CCC TGT GTC |
| **pKS-*SCRE2 -mCherry-NLS* vector construction** | |
| SCRE2-*Hin*d III-F | att aagctt ATG CTC ATC AAC GCC GCC CGC T |
| SCRE2-*Eco*R I-R | att gaattc ATT TTC TTT CCG GCT TGC CTT GCA |
| **pGR107 vector construction of the truncated *SCRE2*** | |
| SCRE2(55-130)-*Xma* I-F | at cccggg ATG CCG CCA GGG GTG AAT CCA TA |
| SCRE2(24-100)-*Xho* I-R | at ctcgag CTA ATA CGA GAG CAG CTT GTC CA |
| SCRE2(1-100)-*Xma* I-F | at cccggg ATG CTC ATC AAC GCC GCC CG |
| SCRE2(55-130)-*Xho* I-R | at ctcgag CTA ATT TTC TTT CCG GCT TGC CTT |
| SCRE2(55-130)-*Xma* I-F | at cccggg ATG CCG CCA GGG GTG AAT CCA TA |
| SCRE2(1-100)-*Xho* I-R | at ctcgag CTA ATA CGA GAG CAG CTT GTC CA |
| SCRE2(55-85)-*Xho* I-R | at ctcgag CTA GAC CGA AAC GCG CGG GCT CG |
| SCRE2(70-100)-*Xma* I-F | at cccggg ATG AAC GGA GAG GAC TGG TGC AA |
| SCRE2(55-80)-*Xho* I-R | at ctcgag CTA GCT CGC GCA GTG CTT GCA CC |
| SCRE2(60-85)-*Xma* I-F | at cccggg ATG CCA TAC AGC TTG ATC GAC ACG C |
| SCRE2(65-85)-*Xma* I-F | at cccggg ATG GAC ACG CTC CTC AAG AAC GG |
| SCRE2(55-85)-*Xho* I-R | at ctcgag CTA GAC CGA AAC GCG CGG GCT CG |
| SCRE2(55-130)-*Xma* I-F | at cccggg ATG CCG CCA GGG GTG AAT CCA TA |
| SCRE2(65-83)-*Sal* I-R | at gtcgac CTA AAC GCG CGG GCT CGC GCA GT |
| SCRE2(68-85)-*Xma* I-F | at cccggg ATG CTC AAG AAC GGA GAG GAC TG |
| SCRE2(70-100)-*Xma* I-F | at cccggg ATG AAC GGA GAG GAC TGG TGC AA |
| SCRE2(70-100)-*Xho* I-R | at ctcgag CTA ATA CGA GAG CAG CTT GTC CA |
| SCRE2(1-80)-*Xma* I-F | at cccggg ATG CTC ATC AAC GCC GCC CG |
| SCRE2(86-130)-*Xma* I-F | at cccggg ATG GAC GCC GGC CGC TAC AAA GC |
| SCRE2(86-130)-*Xho* I-R | at ctcgag CTA ATT TTC TTT CCG GCT TGC CTT |
